# Alterations in Evoked Otoacoustic Emissions by the Use of Meglumine Antimoniate in American Tegumentary Leishmaniasis Patients

**DOI:** 10.1371/journal.pone.0168492

**Published:** 2017-01-03

**Authors:** Débora Cristina de Oliveira Bezerra, Renata Oliveira de Barcelos, Ellen Carvalho de Castro, Claudia Cristina Jardim Duarte, Raquel de Vasconcellos Carvalhaes Oliveira, Tania Salgado de Sousa Torraca, Maria Helena de Araújo-Melo, Frederico Pereira Bom Braga, Benivaldo Ramos Ferreira Terceiro, Lúcia Regina do Nascimento Brahim Paes, Armando de Oliveira Schubach, Cláudia Maria Valete-Rosalino

**Affiliations:** 1 Evandro Chagas National Institute of Infectious Diseases, Oswaldo Cruz Foundation (INI-FIOCRUZ), Rio de Janeiro, Brazil; 2 Department of Otorhinolaryngology and Ophthalmology, Faculty of Medicine, Federal University of Rio de Janeiro, (UFRJ), Rio de Janeiro, Brazil; 3 Department of Otorhinolaryngology, Faculty of Medicine, Federal University of the State of the Rio de Janeiro, (UNIRIO), Rio de Janeiro, Brazil; 4 Brazilian National Council for Scientific and Technological Development (CNPq), Brasilia, Brazil; 5 Carlos Chagas Filho Foundation for Research Support of the state of Rio de Janeiro (FAPERJ), Rio de Janeiro, Brazil; 6 Hygiene and Tropical Medicine Institute (IHMT), New University of Lisbon (UNL), Lisbon, Portugal; Ohio State University, UNITED STATES

## Abstract

**Introduction:**

Tegumentary Leishmaniasis (TL) is a neglected, non-contagious, infectious disease, caused by different protozoa species of the *Leishmania* genus that affects skin and mucous membranes. Meglumine Antimoniate (MA), the first drug of choice for TL treatment in Brazil, has already been associated with cochlear toxicity, which is defined as damages of the cochlea caused by exposure to chemical substances, resulting in reversible or irreversible hearing loss. Auditory monitoring for cochlear toxicity aims at the early detection of auditory disorders, enabling, when possible, hearing to be preserved or an early auditory rehabilitation. Although otoacoustic emissions (OAEs) are used in this monitoring, there is no consensus on the criteria that define cochlear toxicity by this examination. The objective of this study was to describe the characteristics of the OAEs in cochlear toxicity monitoring in TL patients using MA.

**Methods:**

Prospective and longitudinal study of auditory monitoring of 35 patients with parasitological diagnosis of TL, with liminal tonal audiometry, high frequency audiometry, immitanciometry, distortion product evoked otoacoustic emissions (DPEOAEs) and transient evoked otoacoustic emissions (TEOAEs) before treatment, at the end of treatment, one month after the end of treatment and two months after the end of treatment.

**Results:**

80% male, with median age of 44 years (IIQ: 22–59). In the pre-treatment evaluation: 11.4% complained of hearing loss and 20% of tinnitus, 48.6% presented auditory alterations in liminal tonal audiometry (LTA, 65.2% in high frequency audiometry (HFA), 26.6% in DPEOAE and 51.4% in TEOAE. No association was verified between genre and alterations in the EOAE examinations. We observed that patients that presented disorders in DPEOAE examinations were 17 years older than those without alterations and that patients that showed disorders in TEOAEO examinations were 34 years older than those without disorders. The presence of alterations in DPEOAE and TEOAE before beginning treatment was associated with each other and with the presence of alterations in LTA and HFA, and only DPEOAE was associated with hearing loss. We observed a significantly higher number of alterations of DPEOAE at the end of treatment than during pre-treatment and values of the ratio signal/noise significantly smaller at the end of treatment than during pre-treatment in the frequencies of 2 kHz (difference of 1.7dB; p = 0.016) and 4 kHz (difference of 2.45dB; p = 0.016) in DPEOAE and in the range 1.75/2.5 kHz in TEOAE (difference of 2.9dB; p = 0.039).

**Conclusion:**

The ototoxic signals observed in our study using EOAE indicated that both, DPEOAE and TEOAE are adequate and sensitive techniques for clinical monitoring of ototoxicity by MA. Their application is very simple, and their results help the physician to take the most adequate steps for each patient, thus avoiding permanent hearing damage.

## Introduction

Leishmanisis is among the six most important infectious diseases in the world, due to its high rate of detection and the capacity of producing sequelae[1]. Based on estimates, between 0.2 and 0.4 million new cases and between 0.7 and 1.2 million new cases of visceral leishmaniasis and tegumentary leishmaniasis (TL) respectively, occur worldwide every year. The ten countries with the highest TL incidence are: Afghanistan, Algeria, Colombia, Brazil, Iran, Syria, Ethiopia, North Sudan, Costa Rica and Peru. These countries, together, account for 70 to 75% of the estimated global incidence[1,2]. In 2013, 18,226 new cases were reported in Brazil [3]. Notwithstanding this importance, it is still classified as a neglected disease.

TL is a non-contagious, infectious disease, caused by different protozoa species of the *Leishmania* genus that affects skin and mucous membranes. It is transmitted by the bites of female Phlebotomus (Dipteran, Psychodidae, Phlebotominae)[4]. The lesions of cutaneous leishmaniasis (CL) can be single or multiple and occur next to the site of inoculation by the bite of the infected Phlebotomus, after an incubation time that ranges from 15 days to 3 months. The most common form is an ulcerated lesion in areas exposed to the Phlebotomus bite and generally presents raised borders, flat base and granulose surface [5]. The mucosal lesion occurs from blood or lymph spread, weeks or years after the primary skin lesion has healed. In mucosal leishmaniasis (ML) there is a gradual tissue destruction of the upper airways and digestive tract, due to the inflammatory response that can involve nasal and oral mucosa, pharynx and larynx[4].

Pentavalent antimonials (PA) are used for TL treatment and there are two formulations available in the market: meglumine antimoniate (MA) and sodium stibogluconate. The World Health Organization (WHO) preconizes the use of 20mg Sb^5+^/kg/day doses of PA, through intramuscular or intravenous administration, for at least four weeks to treat CL and ML patients. ^1^ In Brazil, the Ministry of Health (MH) recommends the use of MA with doses of 10–20 mg Sb^5+^/kg/day during 20 days to treat patients with CL. ML patients should use 20mg Sb^5+^/kg/day during 30 days, with a maximum daily limit of 3 ampoules for both cases[4].At the Laboratory of Clinical Research and Leishmaniasis Surveillance (LapClinVigileish) of the National Institute of Infectious Diseases (INI)—Fiocruz, an IM dose of 5 mgSb^5+^/kg/day has been effective and well tolerated by TL patients [6,7].

Although PA are the first drug of choice, they cause toxicity and present some adverse effects such as: musculoskeletal pain, gastrointestinal disorders, mild to moderate headache, altered ECG with prolonged QT interval, possible mild to moderate enlargement of the liver and pancreas, nausea, itching at the injection site, generalized itching, myalgia, fever, skin rash, arthralgia, asthenia and local inflammatory reaction [8].

Ototoxicity is described as damage of the cochlea and/or vestibular apparatus caused by the exposure to chemical substances resulting in hearing loss and imbalance, which can be reversible or irreversible [9]. In cochlear toxicity, the onset of auditory symptoms can be slow or treacherous, even after the drug administration is finished. There is usually a direct relationship between the administered dose and the severity of the cochlear lesion [10]. Hearing loss can impair speech understanding, affecting socialization and causing psychosocial damage. [11]. Auditory monitoring for cochlear toxicity aims at the early detection of auditory disorders, enabling when possible, that hearing is preserved or an early auditory rehabilitation. In recent decades, in addition to liminal tonal audiometry (LTA), high frequency audiometry (HFA) and otoacoustic emissions (OAE) have been used for cochlear toxicity monitoring, separately or combined [10, 11, 12]. Criteria to define cochlear toxicity are: increase in LTA ≥25 dB in one frequency or ≥10 dB in two adjacent frequencies or when the high frequencies observed before the beginning of treatment were absent in the subsequent examinations. [13]. However, there is no consensus regarding the criteria that define cochlear toxicity by OAE examination [11].

The first case report of auditory toxicity in leishmaniasis patients using meglumine antimoniate was published recently [14]. From auditory monitoring with liminal tonal and high frequency audiometries of CL patients under treatment with MA, cochlea toxicity was observed in 57.7% cases [15]. However, there are no reports in the literature on alterations in OAE related to MA use. The objective of this research was to describe the characteristics of the otoacoustic emissions in cochlear toxicity monitoring in TL patients using MA.

## Methods

A prospective longitudinal study conducted from March, 2010 to October, 2014, with auditory monitoring through distortion product evoked otoacoustic emissions (DPEOAE) and transient evoked otoacoustic emissions (TEOAE), in patients with parasitological diagnosis of TL by one or more methods (direct examination- scarification or imprint, histopathology, culture, immunohistochemistry or polymerase chain reaction), older than 15 years and treated with intramuscular MA. The study was approved by the Committee on Ethics in Research of the Evandro Chagas National Institute of Infectious Diseases (INI) of the Oswaldo Cruz Foundation (FIOCRUZ), Rio de Janeiro, Brazil and all the patients signed a free and informed consent form. For the children that participated on this study, we obtained the free and informed consent form from their parents or legal representatives.

In the pre-treatment evaluation, patients were submitted to otoscopy, anamnesis of auditory symptoms, immitanciometry, LTA and HFA. All the examinations were conducted in a soundproof booth. Immitanciometry was performed with a ZODIAC 901 (GN Otometrics) immitanciometer, determining static immitance and tympanometry, and classifying the curves into types A, As, Ad, B and C. ATL and AAF were performed with a MADSEN ITERA II (GN Otometrics) audiometer and Sennheiser HDA 200 (SENNHEIER) phones, by determination of aerial auditory thresholds in both ears, with frequencies from 0.25 to 16 kHz. Auditory thresholds above 25 dB in LTA and HFA were considered as hearing loss [16]. In evoked otoacoustic emission testing (EOAE) a MADSEN CAPELLA (GN Otometrics) equipment was used, with platform of Noah software, accepting probe stability above 70%, and with stimulus between 75 and 85 dB NPS. The signal/noise ratio ≥ 3 dB was considered as normal pattern and results below this value were considered as alterations [17]. The frequencies evaluated in DPEOAE testing were 1.25\1.75 kHz, 1.75\2.5 kHz, 2.5\3.5 kHz, 3.5\4.5 kHz. Auditory monitoring with OAE occurred before treatment, at the end of treatment, one month after the end of treatment and two months after the end of treatment. Otoscopy and immitanciometry were used to exclude middle ear involvement during auditory monitoring.

In the exploratory data analysis of categorical variables (gender, complaint of hearing loss, tinnitus, alterations in LTA, alterations in HFA) absolute and relative frequencies were used. The normality of the variables age and signal/noise ratio was rejected by the Shapiro-Wilk test, at 5% significance level. Therefore, the median and the interquartile interval (IIQ) were calculated for those variables. The comparison between the categorical variables was done by Pearson’s chi-squared test or Fisher´s test. The McNemar test was used to compare presence or absence of alterations in DPEOAE and TEOAE before treatment, at the end of treatment, and 1 and 2 months after the end of treatment. The comparison between age and presence of alterations in DPEOAE and TEOAE was done by the Mann-Whitney test. To compare the signal/noise ratio before treatment, with this ratio at the end of treatment and 1 and 2 months after the end of treatment, in DPEOAE and TEOAE examinations, the non-parametric Wilcoxon test was used. The significance level used in the statistical tests was 5%. The analyses were performed with the Statistical Package for Social Sciences—(SPSS) version 16.0 statistical program.

## Results

Of 35 patients monitored, 28 (80%) were male, aged between 16 and 77 years, with median 44 years (IQR: 22–59). Only 11.4% reported complaint of hearing loss before treatment and 20% of tinnitus, while 48.6% patients already presented some auditory alteration in LTA, 65.2% in HFA, 26.6% in DPEOAE and 51.4% in TEOAE. No patient presented compromise of the mid-ear during the auditory monitoring.

No association was verified between gender and alterations in DPEOAE examination (p = 1.0) and TEOAE (p = 0.905) before treatment. However, patients without alteration in DPEOAE were aged less (median = 40 years; IQR = 18–51) than patients with alteration in DPEOAE (median = 57 years; IQR = 46.5–66), p<0.001. Similar behavior was observed when patients without alteration in TEOAE (median = 23 years; IQR = 16.75–44) were compared to those with alterations (median = 57 years; IQR = 44.50–62.75), p<0.001. The alterations in DPEOAE and TEOAE before beginning treatment were associated among each other and with alterations in LTA and HFA and only DPEOAE was associated with complaints of hearing loss; see “Tables 1 and 2”.

**Table 1:** Comparison between presence and absence of alterations in distortion product evoked otoacoustic emissions (DPEOAE) before beginning treatment with Meglumine Antimoniate (MA) of 64 ears of 32 patients with tegumentary leishmaniasis. Evandro Chagas National Institute of Infectious Diseases–Oswaldo Cruz Foundation, 2016.

	Presence of Change in DPEOAE				
	Yes		No		
	(N = 17)		(N = 47)		
Variables prior to initiation of treatment with MA	N	%	N	%	*P*
**Presence of complaints of hearing loss**	4	23,50%	1	2,10%	**0,015**
**Presence of tinnitus**	4	23,50%	6	12,80%	0,435
**Presence of change Audiometry High Frequency**	15	93,80%	24	51,10%	**0,002**
**Presence of Changes in audiometry Conventional**	15	88,20%	14	29,80%	**<0,001**
**Presence of changes of TEOAE**	14	82,40%	16	34,00%	**0,001**

**Table 2:** Comparison between presence and absence of alterations in transient evoked otoacoustic emissions (TEOAE) before beginning treatment with Meglumine Antimoniate (MA) of 70 ears of 35 patients with tegumentary leishmaniasis. Evandro Chagas National Institute of Infectious Diseases–Oswaldo Cruz Foundation, 2016.

Presence of Change in TEOAE					
	Yes		No		
	(N = 36)		(N = 34)		
Variables prior to initiation of treatment with MA	N	%	N	%	*P*
**Presence of complaints of hearing loss**	6	16,70%	1	2,90%	0,107
**Presence of tinnitus**	7	19,40%	3	8,80%	0,308
**Presence of change Audiometry High Frequency**	31	88,60%	14	41,20%	**<0,001**
**Presence of Changes in audiometry Conventional**	24	66,70%	9	26,50%	**0,001**

**Fig 1:**
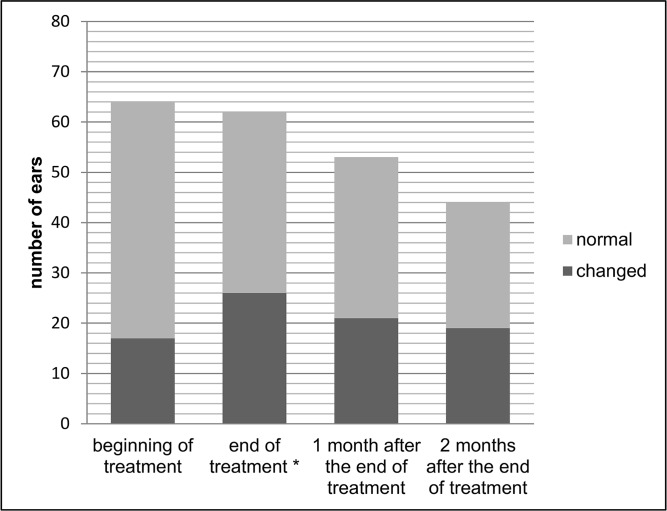
Auditory monitoring with distortion product evoked otoacoustic emissions before treatment, at the end of treatment and 1 and 2 months after the end of treatment of meglumine antimoniate in american tegumentary leishmaniasis patients. * Higher number of alterations in distortion product evoked otoacoustic emissions at the end of treatment than before treatment (p = 0.039).

**Fig 2:**
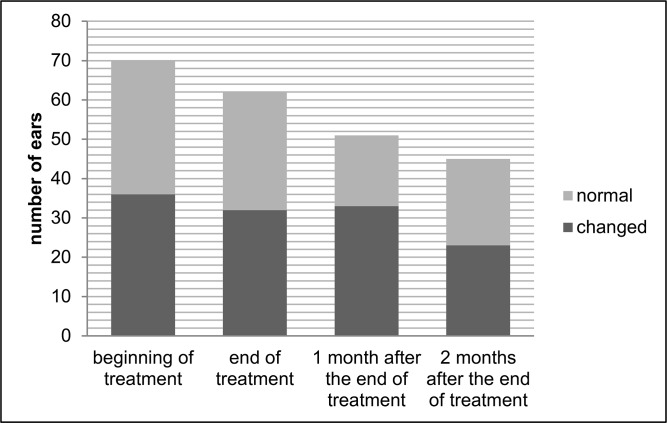
Auditory monitoring with transient evoked otoacoustic emissions before treatment, at the end of treatment and 1 and 2 months after the end of treatment of meglumine antimoniate in american tegumentary leishmaniasis patients.

Figs 1 and 2 show auditory monitoring by DPEOAE and TEOAE testing, before treatment, at the end of treatment and 1 and 2 months after the end of treatment. A significantly higher number (p = 0.039) of alterations in DPEOAE were observed at the end of treatment than before treatment.

**Fig 3:**
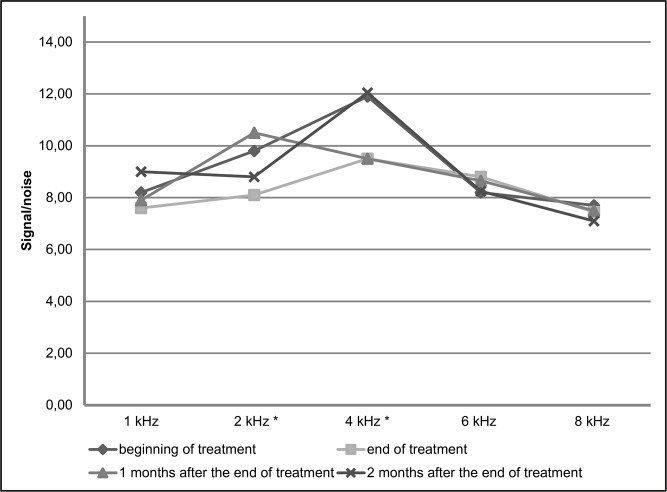
Comparison of the medians of the signal/noise ratio in distortion product evoked otoacoustic emissions before treatment and at the end of treatment, in patients with tegumentary leishmaniasis treated with meglumine antimoniate, Rio de Janeiro, 2016. * signal/noise ratio significantly smaller at the end of treatment than before treatment at the frequencies of 2 kHz (difference of 1.7dB; p = 0.016) and 4 kHz (difference of 2.45dB; p = 0.016)

**Fig 4:**
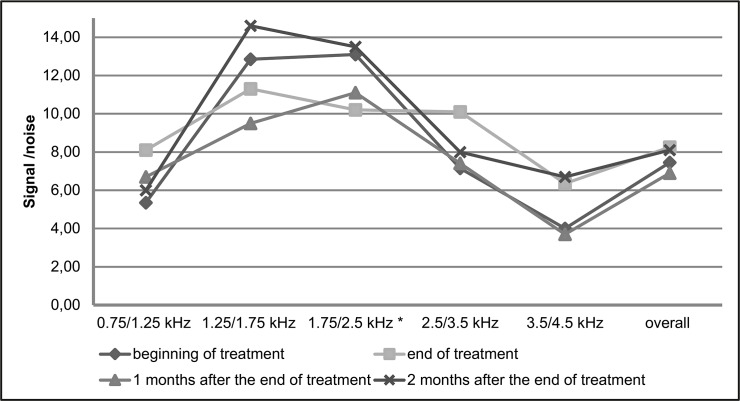
Comparison of the medians of the signal/noise ratio in transient evoked otoacoustic emissions before treatment and at the end of treatment, in patients with tegumentary leishmaniasis treated with meglumine antimoniate, Rio de Janeiro, 2016. * signal/noise ratio significantly smaller at the end of treatment than before treatment in the range 1.75/2.5 kHz (difference of 2.9dB; p = 0.039).

In Figs 3 and 4, the medians of the signal/noise ratio are compared before treatment, at the end of treatment and 1 and 2 months after the end of treatment, in the DPEOAE and TEOAE examinations, respectively. We observe significantly lower values of signal/noise ratio at the end of treatment than before treatment at the frequencies of 2 kHz (difference of 1.7dB; p = 0.016) and 4 kHz (difference of 2.45dB; p = 0.016) in DPEOAE and in the range from 1.75 to 2.5 kHz in TEOAE (difference of 2.9dB; p = 0.039).

## Discussion

This is the first study describing MA effect in EOAE. From auditory monitoring with OEAE of 35 patients with TL under treatment with MA, we verified that at the end of treatment significant alterations of OEAE occur, especially in frequencies of 2 kHz and 4 kHz in DPEOAE and in the range 1.75–2.5 kHz in TEOAE, and that those alterations tend to decline 1 or 2 months after the end of treatment.

We found that patients with alterations in DPEOAE and TEOAE examinations before treatment were 17 years and 34 years older, respectively, than those without alterations. This occurs because, when the age increases, the auditory thresholds tend to increase due to aging of the auditory system and to its continuous exposure to risk factors for hearing loss, such as noise. The older age in TEOAE is justified, because this examination is considered altered in front of any hearing loss, even though mild, while DPEOAE are only absent from a moderate hearing loss [17, 18]. However, we did not find differences related to gender, which has already been described in another study [18].

Complaints of hearing loss were only associated to alterations in DPEOAE. This is also explained because this examination detects moderate or greater hearing losses, when normally the patient becomes aware of his hearing loss. Self-perception of hearing loss seems to be related with the pure-tone average of mid frequencies (1, 2, 3 and 4 kHz), and is more frequent in individuals with moderate to severe hearing loss than among those with mild hearing loss [19]. The TEOAE alter from mild hearing losses, which are usually asymptomatic, we did not observe association with complaint of hearing loss [17] For this reason, although both examinations are associated with alterations in LTA and HFA, the percentage of people with altered LTA among those with TEOAE alteration is lower than among those with DPEOAE alteration.

During auditory monitoring with EAOE, we verified association of alterations in DPEOAE at the end of treatment with the total dose of MA. After the interruption of MA treatment, these alterations tend to regress, as already observed in previous studies, which showed that amplitude reductions in DPEOAE and TEOAE regressed after interrupting treatment with this ototoxic drug [20, 21]. Particularly, MA is a depot drug, with gradual accumulation. The therapeutic effect of antimony is provided by the fraction accumulated in the tissues [22, 23]. PA present an initial absorption phase, followed by a phase of rapid elimination of more than 80% of the administered dose within six to eight hours [4], and finally a slow elimination phase with a half-life of 76 hours [22]. There are *in vivo* evidences of MA conversion into the ionic species Sb^5+^ and Sb^3+^ (*in vivo* active form). Sb^5+^ is excreted faster, because it is free in the plasma, while Sb^3+^ is accumulated in body fluids, suggesting that Sb^3+^ production could be responsible for the prolonged action, whether toxic or therapeutic of the drug [24]. Temporary changes in EOAE and/or in absence of audiometric hearing loss appear to be indicative of sensitivity to pre-clinical damage. Thus, an unknown proportion of false positives can be pre-clinical changes detected by DPEOAE and, as new gold standard methods arise for ototoxicity monitoring, the accuracy of DPEOAE can grow [25, 26]. Although it has been described that cochlear toxicity lesions initially affect high frequencies, which was also described specifically for MA, in a previous study conducted by our group [15], the frequencies that were altered in DPEOAE were 2 kHz and 4 kHz. Although 4kHz and 6kHz are the frequencies that most likely reflect cochlear changes, 2kHz and even 1kHz, can be useful to capture DPEOAE alterations associated with ototoxic medication when the individuals under test have hearing loss previous to the beginning of its use [25], which occurred in our study. Additionally, it was observed that, in patients with hearing loss prior to the start of the ototoxic medication, the frequency that showed greatest alteration of DPEOAE was 4kHz [26]. In this manner, as hearing loss caused by age or other risk factors usually affects high frequencies, it is expected that the amplitudes initially obtained in the EOAE in individuals with prior hearing loss are smaller at these frequencies and, therefore, that it is more difficult to obtain an amplitude reduction that is considered significant. We observed that the frequencies 2 and 4 kHz presented greater amplitudes before the beginning of MA treatment. Thus, it was easier to observe the reduction in those frequencies during ototoxicity monitoring.

While TEOAE evaluate the cochlea only up to 4 kHz and are more sensitive to mild hearing losses, DPEOAE are not so sensitive to mild losses but they evaluate high frequencies [18]. Thus, DPEOAE detect ototoxicity alterations earlier and more often than TEOAE, because they evaluate the highest frequencies, which first alter in ototoxicity and because they can be obtained in the presence of severe hearing losses, making more individuals eligible for auditory monitoring [11, 27]. Therefore, the use of TEOAE has been indicated as the best choice for universal newborn hearing screening, while DPEOAE are prioritized for auditory function monitoring [18], and few studies evaluate TEOAE alterations in ototoxicity [18, 28]. In our study, we observed significant amplitude reduction in EAOET only in the frequency range 1.75–2.5 kHz. In guinea pigs amplitude reductions in the range 2-4kHz, were also observed, although grater in DPEOAE. In addition, considering that a correlation between amplitude reduction in the frequencies of 2, 3 and 4kHz in DPEOAE, with amplitude reduction in the range 2-4kHz in TEOAE [28], has already been established, we can suggest that the alteration we found in the range 1.75–2.5 kHz in TEOAE reflect alterations in the frequency 2kHz in DPEOAE.

Considering that in experimental studies of cisplatin with guinea pigs [28] the amplitude reduction observed both in TEOAE and DPEOAE was associated with severe damage of the organ of Corti, with loss and damage of the hair cells from the basal and middle regions of the cochlea to its apical portion, in addition to serious damage to the stria vascularis, we can assume that those changes may also have occurred in our patients after MA use, because amplitude reduction in both TEOAE and DPEAE implies the involvement of outer hair cells in the ototoxity induced by MA. However, to really know through what mechanism MA is ototoxic, experimental studies are needed.

Considering also that, in ototoxicity, the basal regions of the cochlea are damaged first, leading to high-frequency bilateral symmetrical, sensorineural hearing loss, which progresses to low-frequency hearing loss with higher cumulative doses of certain drugs, finding alterations in the frequencies of social communication (2kHz and 4kHZ) in our study through EOAE, indicates that using these exams in ototoxicity monitoring is adequate. This is because, these alterations indicate that the interruption, at least temporary, of the drug should be considered to allow EOAE return to the normal pattern, as occurred in our study, before definitive injuries with permanent functional impairment occur.

We observed a reduction of the response amplitude of 1.7 dB in 2 kHz and 2.45 dB in 4 kHz in DPEOAE and of 2.9 dB in the range 1.75/2.5 kHz in TEOAE. Significant amplitude reductions of 1dB in 3kHz, 1.15dB in 6kHz and 4.70 in 8kHz, in DPEOAE have also been reported [18]. Although EOAE are increasingly being considered as a key component of auditory monitoring in individuals at risk of ototoxicity, there is no precise definition of how to interpret the results [11, 13, 26, 29]. Some studies have attempted to define reference values of amplitude reductions in DPEOAE that could be clinically used to define ototoxicity. However, most of those studies used a cohort of individuals mostly with normal hearing, or close to normal, making it difficult to generalize those values to individuals that already have some degree of hearing loss before beginning treatment with the ototoxic drug [26]. Although HFA is known to be more sensitive in the early detection of ototoxicity auditory alterations than EOAE [11, 27], both have limitations. Auditory monitoring, both with HFA and EOAE, present limitations in patients with hearing loss, especial in the elderly, because either, no response or reduced responses can be obtained before beginning treatment, due to pre-existing losses of outer hair cells in the basal region of the cochlea. In the same way, HFA and mainly EOAE can be altered due to disease of the middle ear. For this reason it is necessary to include tympanometry in the battery of tests for auditory monitoring, to exclude the impairment of the middle ear that can be responsible for the alterations found in the examinations [11]. Even though the impairment of the middle ear in TL [30] is not as usual as in other situations where ototoxic drugs are employed, such as patients under chemotherapy and with head and neck being irradiated [11], all our patients were evaluated by otoscopy and tympanometry and none presented alterations, turning our monitoring more reliable. Thus, the selection of which examination should be used in auditory monitoring relies on some factors such as age, ability to respond to behavioral tests and the clinical status of the patient [27]. It has been reported that some of the advantages of using EOA is the early detection of ototoxicity and that they are strong predictors of the audiometry alterations determined by ASHA as indicative of cochlear toxicity. Furthermore, it is a non-invasive, simple and objective technique for identifying changes in behavioral auditory thresholds, when the patient is not capable of providing reliable results in hearing tests, due to illness or extreme fatigue, as for example, during chemotherapy treatment [13, 21, 25, 29]. But, when an EOAE alteration is detected in relation to the reference examination, the next step should be initiating a more complete battery of audiometry tests, such as audiometry [26]. When hearing loss is inevitable, the auditory monitoring program should provide the access of the patients to the process of selecting, indicating and adapting an individual hearing aid as well as to rehabilitation programs. [27].

Considering that TL is a neglected disease, and that the use of MA presents numerous toxic effects that put the patient’s life at risk, it is difficult to determine an audiometry monitoring protocol for a patient. Particularly in elderly patients with TL in endemic areas, which may already have a prior presbycusis at the beginning of treatment, the risk of irreversible deterioration of the hearing thresholds for ototoxicity is greater and should be considered, as this may lead these individuals to social isolation. So, further studies should be able to set criteria to establish when the risk of deafness justify a therapeutic change. The ototoxic signals observed in our study, through the use of EOAE, demonstrate that both, DPEOAE and TEOAE are valid and sensitive techniques for clinical monitoring of ototoxicity by MA. Therefore, EOAE may be useful in defining these criteria, since EOAE are a method that is simple to apply and whose results help the physician to take the most adequate steps for each patient, thus avoiding permanent hearing damage.
